# Anti-HER-2 DNA vaccine protects Syrian hamsters against squamous cell carcinomas

**DOI:** 10.1038/sj.bjc.6602853

**Published:** 2005-11-01

**Authors:** G N Berta, B Mognetti, M Spadaro, E Trione, A Amici, G Forni, F Di Carlo, F Cavallo

**Affiliations:** 1 Department of Clinical and Biological Sciences, Ospedale San Luigi Gonzaga, University of Turin, I-10043 Orbassano, Italy; 2 Department of Molecular, Cellular and Animal Biology, University of Camerino, I-62032 Camerino, Italy

**Keywords:** oral squamous cell carcinoma, cancer vaccine, DNA vaccination, HER-2, immunoprevention

## Abstract

This paper illustrates the efficacy of DNA vaccination through electroporation in the prevention of oral transplantable carcinoma in Syrian hamsters. At 21 and 7 days before tumour challenge, 19 hamsters were vaccinated with plasmids coding for the extracellular and transmembrane domains of rat HER-2 receptor (EC-TM plasmids), whereas 19 control hamsters were injected intramuscularly with the empty plasmid. Immediately following plasmid injection, hamsters of both groups received two square-wave 25 ms, 375 V cm^−1^ electric pulses via two electrodes placed on the skin of the injection area. At day 0, all hamsters were challenged in the submucosa of the right cheek pouch with HER-2-positive HCPC I cells established *in vitro* from an 7,12-dimethylbenz[a]anthracene-induced oral carcinoma. This challenge gave rise to HER-2-positive buccal neoplastic lesions in 14 controls (73.37%), compared with only seven (36.8%, *P*<0.0027) vaccinated hamsters. In addition, the vaccinated hamsters displayed both a stronger proliferative and cytotoxic response than the controls and a significant anti-HER-2 antibody response. Most of the hamsters that rejected the challenge displayed the highest antibody titres. These findings suggest that DNA vaccination may have a future in the prevention of HER-2-positive human oral cancer.

Neoplasms of the oral cavity constitute about 3% of all malignancies worldwide (Parkin *et al*, 2001) and their continuous increase to the point of becoming a public health problem in the foreseeable has been predicted by the World Health Organization (http://www.who.int/topics/oral_health/en). Oral squamous cell carcinoma (OSCC) accounts for about 90% of cases. It may be preceded by various lesions and is a complex disease because of its location and patterns of spread. Treatment is essentially determined by site and stage as well as the patient's general health (Vokes *et al*, 1993). Survival rates, however, have not improved over the last 20 years and 50–70% of patients die within 5 years due to local recurrences, metastasis and a second primary cancer (Day and Blot, 1992). Surgery is still the treatment of choice, but often results in chronic pain, speech and swallowing impediments, and irreparable, disfiguring impairments (Mort Telfer and Shepherd, 1993; de Cassia Braga Ribeiro *et al*, 2003).

The alarming epidemiological data, the poor prognosis and the unsatisfactory quality of life of patients with OSCC call for the urgent elaboration of a new way to treat it. Recent preclinical and clinical studies of immunopharmacological methods have shown that they can be effective against melanoma (Nestle *et al*, 1998; Rosenberg *et al*, 1998), lymphoma (Hsu *et al*, 1996), kidney (Kugler *et al*, 2000), prostate (Murphy *et al*, 1999), colorectal (Morse *et al*, 1999) and head and neck cancers (Cortesina *et al*, 1994; Wollenberg *et al*, 1999; De Stefani *et al*, 2002). Anticancer vaccines constitute a specific approach whose efficacy depends on how well the target tumour antigens are defined and whether there are conserved antigens shared by tumours of the same histotype in many individuals (Gilboa, 2004). The literature shows that HER-2 is one of the most common oncogenes overexpressed in OSCC (Chen *et al*, 2003). It encodes for a tyrosine-kinase receptor (p185) belonging to the family of epidermal growth factor receptors. Increased expression or mutation of p185 induces a proliferation signal in a ligand-independent way. p185 is therefore believed to be involved in the triggering and progression of many epithelial tumours (Ignatoski *et al*, 1999). Impressive results have been achieved by targeting p185, particularly in transgenic mice that inevitably develop spontaneous tumours. DNA vaccination with plasmids coding for the transmembrane and extracellular domains of the rat p185 (EC-TM plasmids) elicits a protective immune response against spontaneously arising mammary tumours in BALB/c mice transgenic for the gene encoding the mutated form of the rat p185 (Rovero *et al*, 2000; Di Carlo *et al*, 2001; Dela Cruz *et al*, 2003; Quaglino *et al*, 2004a). This form of vaccination has many ideal features: induction of both humoral and cellular immune responses, long-lasting immunity and simple and cheap production. The first generation of experiments have shown that delivery of DNA constructs encoding a specific immunogen safely elicits well-tolerated responses in a variety of animal models (Lowrie, 2003). Even better results are obtained when the entry of DNA into target cells is facilitated by electroporation, a technique in which short high-voltage pulses are used to form pores in the cell membrane (Gehl, 2003). This paper illustrates the efficacy of DNA vaccination through EC-TM plasmid electroporation (EC-TM DNA-vax) in the prevention of oral cancer in an orthotopic Syrian hamster model of transplantable tumour. The OSCC line (HCPC I) injected into hamster cheek pouches was established from an epidermoid carcinoma induced through *in vivo* applications of 7,12-dimethylbenz[a]anthracene (Salley, 1954; Odukoya *et al*, 1983). Our findings indicate that EC-TM DNA-vax may have a future in the treatment of HER-2-positive human oral cancer, especially in the prevention of its development after surgical removal of an initial OSCC or as the sequel of a precancerous lesion.

## MATERIALS AND METHODS

### Cell lines

The HCPC I line established from a chemically induced epidermoid carcinoma of the Syrian hamster (Odukoya *et al*, 1983) was kindly provided by Professor DT Wong (UCLA, Los Angels, CA, USA). N202.1A and N202.1E, which are positive and negative for rat HER-2 expression, respectively, are clonal derivatives of the established N202.1 cell line derived from a mammary carcinoma of FVB-neuN#202 mice, transgenic for the rat neu proto-oncogene (Nanni *et al*, 2000). SKBr3 (American Type Culture Collection, Manassas, VA, USA) is a human breast cancer cell line that overexpresses the HER-2 gene product. Wild-type BALB/c 3T3 fibroblasts and BALB/c 3T3 fibroblasts stably cotransfected with the wild-type rat HER-2/neu and mouse class I H-2K^d^ and B7.1 genes (BALB/c 3T3-NKB cells) (Spadaro *et al*, 2005) were kindly provided by Dr Wei-Zen Wei (Karmanos Cancer Institute, Detroit, MI, USA). Cells were cultured in DMEM (Sigma-Aldrich, St Louis, MO, USA) with 75 U ml^−1^ penicillin G, 100 *μ*g ml^−1^ kanamycin sulphate and 1 *μ*g ml^−1^ amphotericin B (all from Sigma) at 37°C in a humidified 5% CO_2_ atmosphere. For HCPC I and SKBr3, the medium was supplemented with 10% fetal calf serum (FCS) (Cambrex, Walkersville, MD, USA), whereas for N202.1A, N202.1E, BALB/c 3T3 and BALB/c 3T3-NKB cultures it was supplemented with 20% FCS. Neomycin (600 *μ*g ml^−1^) and Zeocin (600 *μ*g ml^−1^) (Invitrogen Corp., Carlsbad, CA, USA) were added to the BALB/c 3T3-NKB cell medium.

### Immunoprecipitation and Western blot analysis

HCPC I, N202.1A and N202.1E cell monolayers were collected and prepared by homogenisation in protein loading buffer. Total proteins (20 *μ*g) were separated on an 8% SDS–polyacrylamide gel, and electrotransferred to a Hybond-C nitrocellulose membrane (Amersham Pharmacia Biotech, Little Chalfont, UK). Western blot analysis was performed with the mouse monoclonal antibody (mAb) Ab-3 (Oncogene Research Products, Cambridge, MA, USA). The membrane was then exposed to the appropriate horseradish peroxidase-conjugate secondary antibody (1 : 5000) (Sigma) for 1 h at room temperature and labelled bands were detected with a chemiluminescence commercial kit (Immun-Star™, Bio-RadHercules, CA, USA). Image acquisition of the immunoreactive bands was performed with a Kodak Image Station 440CF (Kodak, Rochester, NY, USA).

Immunoprecipitation of tumour, healthy tissue and HCPC I was performed by incubating samples with the rabbit polyclonal antibody Sc-284 (Santa Cruz Biotechnology, Santa Cruz, CA, USA) and with Protein A-sepharoses (Sigma), equilibrated in lysis buffer and conjugated with anti-rabbit IgG (Sigma). After immunoprecipitation, immune complexes were washed, eluted, heated for 5 min at 95°C in reducing sample buffer and processed as described for total samples. Immunoprecipitated proteins were revealed with a pool of DNA-vax or control hamster sera diluted 1 : 10, and the secondary antibody peroxidase-conjugated AffiniPure Rabbit anti-Syrian hamster IgG (Jackson ImmunoResearch, West Grove, PA, USA).

### Flow cytometry

HCPC I cells were stained with Ab-3, which recognises an intracellular domain of p185; SKBr3 cells were used as a control of high Her-2 expression. The anti-mouse FITC-conjugated secondary antibody (1 : 5000) (Dako, Glostrup, Denmark) was used to detect bound primary antibodies. Before staining, the cells were permeabilised with phosphate-buffered saline (PBS) supplemented with 1% FCS and 0.5% Tween 20. Analysis was performed with a FACscan (Beckton Dickinson, Mountain View, CA, USA) and the cells were gated by size and granularity. The data were analysed through CellQuest (Beckton Dickinson).

### Hamsters and HCPC I cell challenge

Thirty-eight 4–6-week-old male Syrian hamsters (Charles River Laboratories Italy, Calco, Italy) were maintained in specific pathogen-free conditions with a 12 h light–dark cycle and rodent chow and tap water *ad libitum*. They were challenged (day 0) with 1.8 × 10^7^ HCPC I cells in 0.2 ml of PBS injected with a 20-gauge needle syringe in the submucosa of the everted and stretched out right cheek pouch. They were then checked weekly to monitor lesion progression and, 5 weeks from the beginning of the experiments, anesthetised, bled and killed. The spleen was collected for the cytotoxicity assay, while all the main organs were inspected macroscopically. All procedures were carried out in accordance with the Guidelines for the Welfare of Animals in Experimental Neoplasia (UKCCCR, 1998).

### Injection and electroporation of EC-TM plasmids

pcDNA3 vector coding the extracellular and transmembrane domains of rat HER-2 receptor was produced and used as described (Rovero *et al*, 2000). Briefly, DNA was precipitated, suspended in sterile saline at 1.25 mg ml^−1^, and stored in aliquots at −20°C for use in immunisation protocols. For DNA electroporation, 50 *μ*g of EC-TM or empty plasmids in 40 *μ*l of 0.9% NaCl with 6 mg ml^−1^ polyglutamate were injected into the tibial muscle of both legs of anesthetised hamsters 21 and 7 days before tumour challenge (day 0). Electric pulses were applied by two electrodes placed on the shaved skin of the injection area covered with a conducting gel. Two square-wave 25 ms, 375 V cm^−1^ pulses were generated by a T820 electroporator (BTX, San Diego, CA, USA).

### Histology and immunohistochemistry

Tissue samples and cells were fixed and embedded in paraffin. Sections (4 *μ*m) were cut and stained with haematoxylin and eosin or analysed for expression of HER-2 using the Sc-284 Ab, or of pancytokeratins with the C-1801 mouse mAb (Sigma). The binding of Sc-284 on fixed samples was evaluated with a Dako En Vision™ Plus System (Dako Corporation, Carpinteria, CA, USA). Peroxidase activity was displayed by 3–3′-diaminobenzidinetetrahydrochloride (Dako Corporation) and a haematoxylin solution was used for nuclear staining. Paraffin sections from mammary carcinomas with known HER-2 expression were used as positive controls. Staining was graded from 0 (negative) to 3 (strong complete membrane staining) according to the manufacturer's instructions (Dako Hercept Test, 2000). On fresh cells growing on a Lab-Tek chamber slide (Nalge Nunc Int, Naperville, IL, USA), after nuclear staining with propidium iodide, Sc-284 and C-1801 binding was demonstrated with the appropriate FITC-conjugated Ab. Images were captured with an Axiovert Zeiss microscope (Zeiss, Axiovert 100 M).

### Cellular reactivity

In the proliferative assay, 4 × 10^5^ spleen cells (Spc) from control and EC-TM DNA-vax hamsters were cultured with 2 × 10^4^ HCPC I cells in 200 *μ*l of medium supplemented with 10 U ml^−1^ of recombinant IL-2 (Eurocetus, Milan, Italy) in quadruplicate in microtitre wells at 37°C in a humidified 5% CO_2_ atmosphere. After 24 h, cells were pulsed for 48 h with BrdU labelling solution. BrdU uptake was detected with the Cell Proliferation ELISA BrdU Kit (Roche Diagnostic GmbH, Mannheim, Germany) and expressed as optical density (OD). To assess cytotoxicity, 1 × 10^7^ Spc were stimulated for 6 days with 5 × 10^5^ mitomycin-C (Sigma)-treated HCPC I cells in the presence of 10 U ml^−1^ IL-2 and assayed in a 48 h [^3^H]TdR release assay at effector-to-target ratios from 50 : 1 to 6 : 1 in round-bottom, 96-well microtitre plates in triplicate, as previously described in detail (Cappello *et al*, 2003). The results were expressed both as percentage of cytotoxicity and as lytic units (LU)_20_ per 10^7^ effector cells, with LU_20_ defined as the number of effector cells needed to kill 20% of the target cells (Cavallo *et al*, 1992).

### Antibody response

Sera obtained at progressive time points from control and EC-TM DNA-vax hamsters were diluted 1 : 100 in PBS-azide-bovine serum albumin (Sigma), and the presence of anti-p185 Ab was determined by flow cytometry using BALB/c 3T3-NKB cells. FITC-conjugated goat anti-hamster Ab specific for hamster IgG (heavy and light chains) (ImmunoKontact, Abingdon, Oxon, UK) was used to detect bound primary Ab. Normal hamster serum was the negative control. mAb Ab-4 (Oncogene) was used as a positive control. Serial Ab-4 dilutions were used to generate a standard curve to determine the concentration (*μ*g ml^−1^) of anti-p185 Ab in serum (Spadaro *et al*, 2005).

### Statistics

Differences in tumour numbers were evaluated using Fisher's exact test. Data on cytotoxicity, lymphocyte proliferation and antibody level were evaluated with a two-tailed Student's *t*-test.

## RESULTS

### HER-2 expression by HCPC I cells

**Figure 1:**
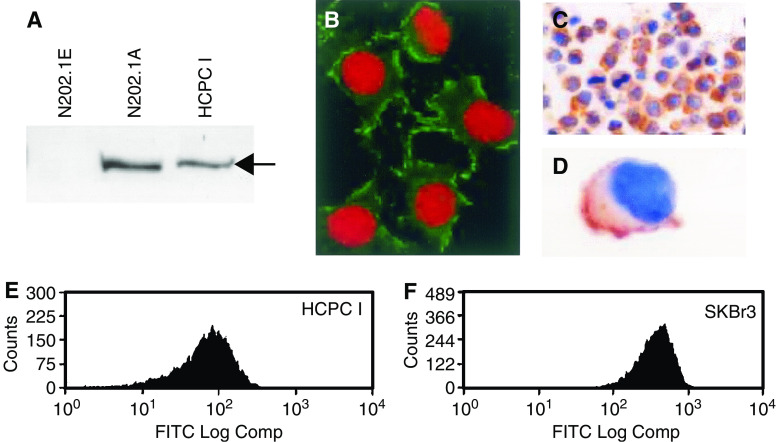
p185 expression by HCPC I. (**A**) Western blot analysis revealed by Ab-3: N202.1E is the negative control, N202.1A the positive control; the arrow indicates a molecular weight of 185 kDa; (**B**) Sc-284 p185 localisation in adherent HCPC I shown by green fluorescent immunostaining. Nuclei are stained red. (Original magnification: × 600). (**C, D**) Immunocytochemical expression of p185 by HCPC I revealed by Sc-284. A predominant membrane staining is shown (original magnification: **C** × 200; **D** × 1000). (**E, F**) FACs analysis performed with mAb Ab-3. In (**E**) HCPC I staining is shown, and in (**E**) SKBr3 are used as a positive control.

As shown by Western blot analysis with mouse N202.1A and N202.1E cells as HER-2-positive and -negative controls, HCPC I cells express p185 receptor (Figure 1A).

Immunostaining of adhering cells (Figure 1B) showed a predominant cell membrane p185 expression; immunohistochemistry of paraffin-embedded HCPC I (Figure 1C, D) showed a medium-high p185 expression (2+) in almost all cells (as compared with breast carcinoma specimens, not shown, according to the manufacturer's instructions) (Dako Hercept Test, 2000). After staining the cells with Ab-3 recognising p185, flow cytometry showed that HCPC I cells were HER-2 positive (Figure 1E). SKBr3, which are known to highly overexpress HER-2, were used as a positive control (Figure 1F).

### Growth of HCPC I tumour cells in Syrian hamsters

**Figure 2:**
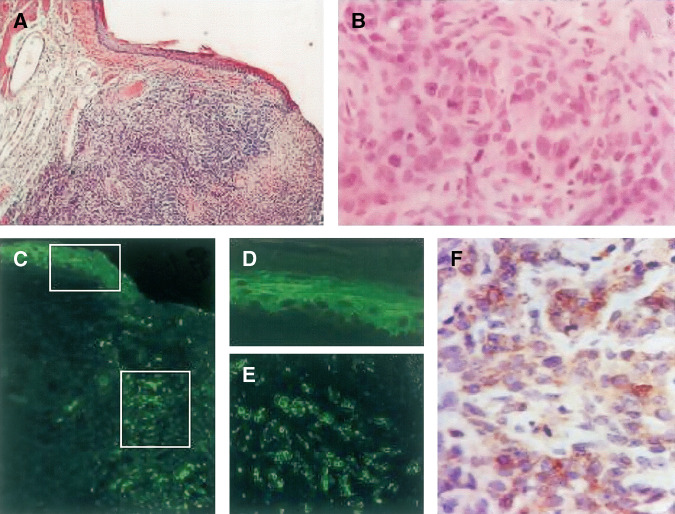
Histology and immunohistochemistry of neoplastic lesions induced in hamster cheek pouches by HCPC I. (**A**) Undifferentiated SCC (× 25). At higher magnification (**B**, × 400), cellular pleomorphism, numerous mitoses and large hyperchromatic nuclei are present in elongated neoplastic cells. Haematoxylin and eosin staining. Indirect immunofluorescent staining for pancytokeratin confirms the epithelial origin of the tumour infiltrating cells (**C**, × 25, **E**, × 200). The positive control is the normal epithelium (**D**, × 200). p185 expression is maintained in inoculated HCPC I cells with a predominant peripheral or membrane cell distribution (**F**, immunoperoxidase, × 400).

At 5 weeks after HCPC I cell challenge, 14 out of 19 hamsters (73.37%) injected with the empty plasmid 21 and 7 days before challenge displayed buccal lesions (mean 3–11 mm). Six hamsters presented small nodules, three of which were associated with mucosal ulceration, while eight showed irregularly shaped white plaques. Irrespective of the gross appearance, all lesions had the microscopical features of invasive squamous carcinoma. The lesions were characterised by solid nests and cords composed of large roundish or, more commonly, elongated neoplastic cells, with occasional atypias, an hyperchromatic nucleus and numerous mitoses, extensively infiltrating the wall of the pouch mucosa and underlying striated muscle fibres. In some cases, the overlying mucosa was invaded and ulcerated. Keratin pearls and multinucleated cells were occasionally observed. Foci of necrosis were present in most cases (Figure 2A and B). The epithelial origin of the infiltrating cells was confirmed by immunohistochemical expression of cytokeratin (Figure 2C, D and E). The tumour cells were also focally positive for HER-2, with preferential peripheral or membrane staining (Figure 2F). In the five tumour-free hamsters, the whole tissue challenge areas displayed only an inflammatory and fibrotic reaction. A dense fibrous tissue nodule containing occasional inflammatory cells, but no tumour cells (as confirmed by negative cytokeratin staining) was seen in two hamsters (data not shown).

### Anti-HER-2 DNA vaccine protects against HCPC I cell challenge

Only seven out of 19 (36.8%, *P*<0.0027) hamsters vaccinated with EC-TM plasmids 21 and 7 days before challenge displayed a neoplastic lesion. Five displayed a nodular mass, the other two a white plaque only. The histological features of these lesions were similar to those of the controls. No signs of acute toxicity or weight differences were associated with EC-TM DNA-vax.

### Cell reactivity

**Figure 3:**
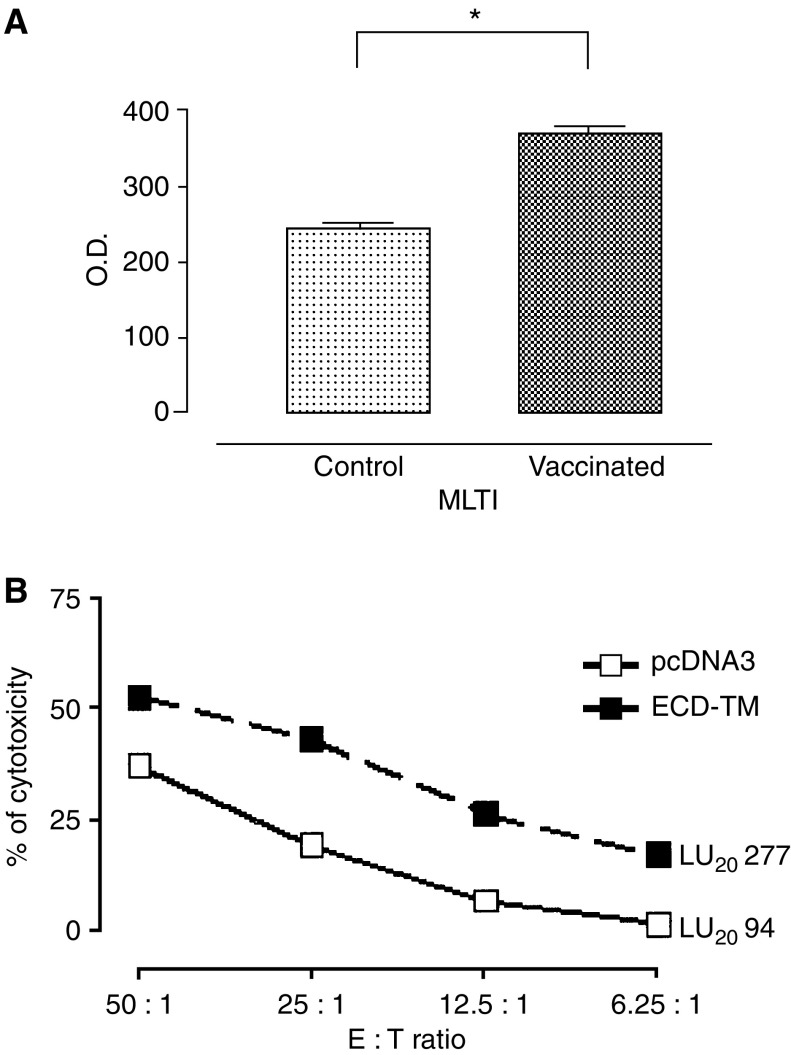
Cellular reactivity. (**A**) Proliferative response. Spc were restimulated for 48 h with HCPC I cells in the presence of BrdU. Results are from one of three independent experiments in which each sample was prepared in triplicate (^*^P<0.05). (**B**) Cytotoxicity. Spc cytotoxicity evaluated in a 48 h [^3^H]thymidine release assay against HCPC I (filled squares, hamsters vaccinated with EC-TM plasmids; empty squares hamsters vaccinated with empty plasmid) (*P*=0.009). Data from an experiment representative of three similar experiments are shown.

To see whether enhanced cell reactivity was associated with the protection afforded by EC-TM DNA-vax, the proliferative and cytotoxic response of Spc from EC-TM-vaccinated and control hamsters to HCPC I cells was assessed at the end of the experiment. Following *in vitro* restimulation, Spc from EC-TM-vaccinated hamsters displayed a stronger proliferative (Figure 3A) and cytotoxic response (Figure 3B) than those from control hamsters.

### Anti-HER-2 antibodies

**Figure 4:**
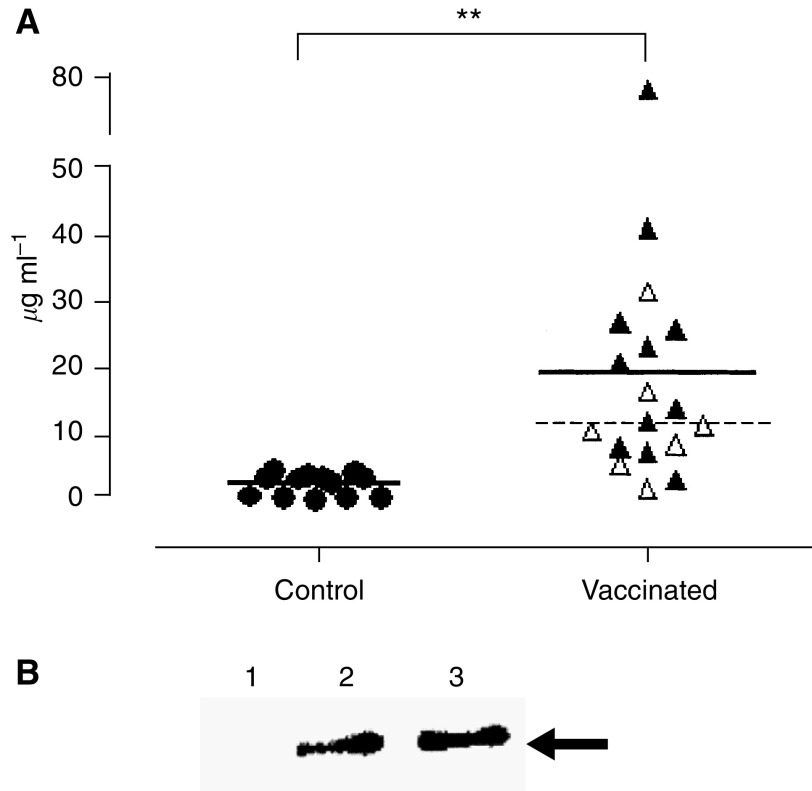
Specific antibodies against p185. (**A**) Anti p185-antibody titre in vaccinated and control animals. Mean antibody titres are indicated by full line (^**^P=0.0024). Antibody titre of EC-TM DNA-vax hamsters is correlated to vaccination outcome: black and empty triangles represent tumour-rejecting and tumor-bearing animals, respectively. Only two out of seven tumour-bearing hamsters displayed an antibody titre above 11.5 *μ*g ml^−1^ (dotted line). (**B**) Extracts of healthy mucosa (lane 1), tumour tissue (lane 2) and HCPC I (lane 3) immunoprecipitated by Sc-284 and revealed with pooled sera from DNA-vax hamsters. The arrow indicates a molecular weight of 185 kDa.

A significant anti-HER-2 antibody response was detected in sera collected the day before HCPC I challenge from the immunised hamsters. Most of the hamsters that rejected the challenge displayed the highest antibody titres (Figure 4A). These titres were much the same 5 weeks later.

The specificity of those antibodies was confirmed by Western blot. After immunoprecipitation, sera from DNA-vax hamsters recognised the p185 expressed by HCPC I and by tumour tissue but not from healthy controlateral pouch mucosa (Figure 4B). No staining was obtained by sera of control hamsters (data not shown).

## DISCUSSION

This is probably the first demonstration that a protective immune response against HER-2 can be elicited in hamsters by EC-TM DNA-vax as in mice transgenic for the rat HER-2 that develop mammary carcinomas (Rovero *et al*, 2000; Quaglino *et al*, 2004a). Electroporation of plasmids coding for the EC and TM portion of rat HER-2 elicits both T-cell reactivity and antibody response. The direct correlation between the anti-HER-2 Ab titre and rejection of HCPC I suggests that antibodies make a substantial contribution to this protection as in HER-2 transgenic mice (Rovero *et al*, 2000; Nanni *et al*, 2004; Park *et al*, 2005).

Plasmids coding rat HER-2 were employed because xenogeneic immunisation is an effective way to break tolerance and induce T cells and antibodies crossreacting with the self, tolerated protein (Bowne *et al*, 1999; Overwijk *et al*, 1999). Hamster and rat HER-2 proteins share 94.2% sequence similarity (Swiss Prot; www.expasy.org) and long homologous sequences are intercalated with heterologous amino-acid sequences. T-helper cell reactivity against non tolerated rat peptides assists activation of the B cells that will produce antibodies against both rat and hamster HER-2 (Trojan *et al*, 2001). Moreover, in the nine fragments recognised as the most immunogeneic of the EC and TM domains according to Ogiso *et al* (2002), six out of 10 substitutions are conservative. In addition, xenogeneic sequences may give rise to ‘heteroclitic’ peptides that may have a higher affinity for hamster MHC glycoproteins than homologous peptides (Dyall *et al*, 1998).

Vaccination with DNA coding for rat HER-2, in fact, elicits a significant cell (*P*=0.009) and antibody (*P*=0.0024) reaction against hamster HER-2. The absence of obvious autoimmune lesions may be due to poor expression of HER-2 by adult hamster tissues.

The appearance of a significant anti-HER-2 antibody response following electroporation points to T-helper and B-cell activation. Previous work with transgenic mice has illustrated the fundamental role of antibodies in checking the progression of HER-2 carcinoma (Rovero *et al*, 2000; Nanni *et al*, 2004; Quaglino *et al*, 2004a; Park *et al*, 2005). An arbitrary threshold of 11.5 *μ*g ml^−1^ can be used to divide the vaccinated hamsters into those with high and those with low anti-HER-2 antibody titres. Tumour takes were higher in the low-titre hamsters (Figure 4A). The antitumour effects of antibodies directed against membrane tyrosine kinase receptors such as p185 include the blockade of mitogenic signal transduction through inhibition of receptor dimerisation and induction of internalisation and recycling, along with immune-mediated functions, namely complement-mediated cytotoxicity and antibody-dependent cellular cytotoxicity (Rovero *et al*, 2000; Nanni *et al*, 2004; Quaglino *et al*, 2004b). Although transplantable tumours may not reflect a few features of the human cancer, we studied HCPC I cells because the biological and histological characteristics of the tumours they form in hamsters are actually very similar to human SCC. In addition, they are from one of the best-characterised animal models for oral cancer, overexpresses p185 receptors and are transplantable to immunocompetent random-bred hamsters. This is probably the first demonstration of the efficacy of specific immunisation in the control of OSCC. Its significance is enhanced by the fact that a high percentage of OSCC overexpresses p185 receptors (Xia *et al*, 1999; Chen *et al*, 2003) and its extension, via a similar protocol, to another kind of cancer, of the evidence of the ability of DNA electroporation to evoke an efficient immune response to prevent mammary carcinoma in HER-2 transgenic mice (Quaglino *et al*, 2004a). Hamsters are naturally tolerant to the self-endogenous p185 protein and DNA electroporation overcame such tolerance. The hamster cheek pouch is an ideal site for the implantation of tissues and cells, both from other species and humans (Lutz and Patt, 1952; Handler *et al*, 1956; Resnick *et al*, 1960), since it is not an immunologically privileged site and immunosuppressive and immunostimulating agents can modulate the intensity of the local immune response (Shklar *et al*, 1979). Transplantability of HCPC I cells to random-bred hamsters rests on the monomorphic aspect of their major histocompatibility complex (Billingham and Hildemann, 1958; Palm *et al*, 1967). This peculiarity serves to illustrate the efficacy of DNA vaccination against HER-2 in a genetically heterogeneous (not inbred) population displaying some features similar to those in human. This heterogeneity may account for the much higher dispersion of the anti-HER-2 Ab titres after DNA electroporation by comparison with inbred mice (Rovero *et al*, 2000; Quaglino *et al*, 2004a, 2004b).

As no major toxic effects were associated with DNA electroporation, or with the stimulation of a significant anti-HER-2 immune response, the use of DNA electroporation could be considered in OSCC prevention, especially the predominantly local and regional recurrences noted in OSCC patient. Furthermore, it is a relatively low-cost technology that could also be afforded by developing countries, such as India, Bangladesh and Sri Lanka with their high incidence of OSCC and prevalence of HER-2-positive OSCC due to the consumption of betel quid (Chen *et al*, 2003).
